# Chitotriosidase Activity Is Counterproductive in a Mouse Model of Systemic Candidiasis

**DOI:** 10.3389/fimmu.2021.626798

**Published:** 2021-03-16

**Authors:** Nicholas A. Schmitz, Ritesh P. Thakare, Chun-Shiang Chung, Chang-Min Lee, Jack A. Elias, Chun Geun Lee, Brian W. LeBlanc

**Affiliations:** ^1^Division of Surgical Research, Department of Surgery, Rhode Island Hospital, Providence, RI, United States; ^2^Molecular Microbiology and Immunology, Brown University, Providence, RI, United States

**Keywords:** neutrophil (PMN), Candida, candidiasis, chitotriosidase (CHIT1), integrins, clustering

## Abstract

Mammalian cells do not produce chitin, an insoluble polymer of N-acetyl-D-glucosamine (GlcNAc), although chitin is a structural component of the cell wall of pathogenic microorganisms such as Candida albicans. Mammalian cells, including cells of the innate immune system elaborate chitinases, including chitotriosidase (Chit1), which may play a role in the anti-fungal immune response. In the current study, using knockout mice, we determined the role of Chit1 against systemic candidiasis. Chit1-deficient mice showed significant decrease in kidney fungal burden compared to mice expressing the functional enzyme. Using *in vitro* anti-candidal neutrophil functional assays, the introduction of the Chit1:chitin digestion end-product, chitobiose (N-acetyl-D-glucosamine dimer, GlcNAc2), decreased fungal-induced neutrophil swarming and Candida killing *in vitro*. Also, a role for the lectin-like binding site on the neutrophil integrin CR3 (Mac-1, CD11b/CD18) was found through physiological competitive interference by chitobiose. Furthermore, chitobiose treatment of wild type mice during systemic candidiasis resulted in the significant increase in fungal burden in the kidney. These data suggest a counterproductive role of Chit1 in mounting an efficient anti-fungal defense against systemic candidiasis.

## Introduction

Systemic candidiasis continues to be a significant medical problem in critically ill patients with high morbidity and mortality despite available anti-fungal therapy (1). Increasingly complex surgical procedures have led to the increased use of invasive measures such as intravenous catheters and intravenous hyperalimentation that are also risk factors for development of candidiasis (2, 3). Neutrophils are the major effector immune cells in fungal killing, where the mechanism for the killing of *C. albicans* was found to be dependent on the β2 integrin, complement receptor 3 (CR3) (4–8).

CR3 has two spatially distinct binding sites, the so-called I-domain (binds extracellular matrix) and the lectin-like domain (binds fungal β-glucan), that the simultaneous binding of both sites with ligand results in enhanced anti-microbial cellular responses including neutrophil swarming (9–12). Furthermore, neutrophilic recognition of the fungal pathogen *C. albicans* causes the release of anti-microbial molecules from granules at the site of fungal infection including the chitin digestion enzyme chitotriosidase (Chit1), which acts on the non-reducing end of the chitin microfibril, releasing copious N-acetyl-D-glucosamine (NADG) dimer, chitobiose, one by one from the chitin chain (13, 14). Although human cells do not produce chitin, they are exposed through contact with chitin containing pathogens including *Candida albicans*. There are currently two identified human chitinase proteins that are enzymatically active, acidic mammalian chitinase (AMCase) and Chit1 (15). Recent studies have linked chitotriosidase with anti-*Candida* activities however whether it is necessary to mount an effective anti-fungal inflammatory response has not been sufficiently addressed (14, 16–20).

Previous studies have shown that NADG inhibits CR3 activity by competitive interference at its lectin-like site (21, 22). Additionally, the enzymatic activity of Chit1 on fungally-derived chitin can increase the concentration of chitobiose at the site of a fungal infection, specifically at the neutrophil:*Candida* interface. Therefore, the hypothesis to be tested is that Chit1-generated enhanced chitobiose concentration provides an altered microenvironment that interferes with normal CR3-mediated anti-microbial functions resulting in reduced fungal clearance. In this study, we used mice genetically deficient in Chit1 to determine its necessity against systemic candidiasis. Likewise, we determined the effect of chitobiose on CR3-mediated neutrophil anti-fungal functions such as clustering/swarming and *Candida* killing.

## Materials and Methods

### Animals

Sex-matched, 2-mo-old wild type (WT) and chitotriosidase-deficient mice (Chit1^−/−^) were provided by laboratories of Drs. Jack A. Elias and Chun Geun Lee (Brown University) (23). The Chit1^−/−^ mice appeared normal on gross physical and light microscopic examination. C57BL/6 male and female mice (WT), aged 8–12 wks, were obtained from the Jackson Laboratory (Bar Harbor, ME) and used in the chitobiose experiments. All animal protocols were carried out in accordance with the NIH Guide for the Care and Use of Laboratory Animals and were approved by the Rhode Island Hospital Institutional Animal Care and Use Committee (AWC 5020-17).

### Systemic Candidiasis

Mice were injected via the lateral tail vein with 100 μl sterile PBS containing 1 × 10^5^ *C. albicans* (SC5314, ATCC, Manassas, VA) blastoconidia (isolated from culture grown in YEPD media (BD Bioscience, Woburn, MA) overnight at 37°C with shaking) alternating injection between treatment groups. Weights were measured daily until the end of the experiment (Day 5 post injection). At end of experiment, whole blood and tissues were excised and collected for further analysis. Chitobiose or lactose (control) groups were treated with 100 mg/kg disaccharide dissolved in sterile saline and were injected i.p. twice daily for the duration of the experiment.

### Histology

Blinded histological analysis of cryosectioned and stained (both PAS and H&E stains were used) kidney tissue was performed for each mouse utilized in the study. The analysis consisted of five individual categories (inflammatory cells, inflammatory abscesses, tissue injury, and hemorrhage) that were independently scored on a scale of 0–3 by a blinded pathologist. The scoring criteria for each category were as follows: **Inflammatory cells**: 0—No leukocyte infiltration, 1—Mild leukocyte infiltration, 2—Moderate leukocyte infiltration, 3—Severe leukocyte infiltration, **Inflammatory abscesses**: 0—No inflammation present, 1—Mild inflammation covering < of the area of the tissue, 2— Moderate inflammation covering between and of the area of the tissue, 3— Severe inflammation covering > of the area of the tissue, **Tissue Injury**: 0—No parenchymal damage, 1—Mild parenchymal damage, 2—Moderate parenchymal damage, 3—Severe parenchymal damage, **Hemorrhage**: 0—No blood present, 1—Mild amount of blood present, 2—Moderate amount of blood present, 3—Severe amount of blood present. **Overall Histology score** is the sum of all histological values. The resulting inflammatory scores are displayed as averages from data acquired from 11 animals per group.

### Quantification of Neutrophil, Macrophage and Hyphae by Immunohistochemistry

Immunofluorohistological analysis of cryosectioned kidney tissue was performed for each mouse utilized in this study. Kidney sections (10 μm) were adhered to glass slides and blocked with normal goat serum. Sections were stained for neutrophils (Ly6G, Biolegend, rat IgG, 1:100) or macrophages (F4/80, Abcam, rat IgG, 1:100), and hyphae (BfDIV, generous gift of anti-β-glucan antibody from Dr. Jonathan Reichner, mouse IgM, 1:1000) overnight at 4°C. Slides were washed with PBS and probed with goat anti-rat IgG (Invitrogen, Alexa Fluor 488 labeled 1:1000 and goat anti-mosue IgM, Alexa Fluor 647 labeled, 1:1000), mounted with DAPI (Vectashield, Vector Lab), visualized with a Nikon Eclipse Fluorescent microscope (20X objective), and five images/slide were captured using a Qiacam CCD camera. Supplementary Figure 4 (with 4X images showing Ly6G distribution, black outline) shows general area of quantitative (20X) image placement to include areas of active infection (red outline). Mean fluorescent intensity for Ly6G, F4/80 and BfDIV was measured using ImageJ (NIH v1.48v) in five predetermined non-redundant boxes (400 × 400 pixel) within each picture (final value is average of 25 fields per animal normalized by subtracting the fluorescence average of 25 fields from images from immediate serial sections treated with secondary antibody alone (no primary antibody).

### Analysis of Cytokine, MPO, and BUN Concentrations

A multi-plex mouse-specific cytokine/chemokine LEGENDplex bead array (BioLegend) was used according to the manufacturer's protocol on homogenized kidney samples. All samples were run on a Miltenyi MACSquant flow cytometer, and analysis was performed using LEGENDplex analysis software (BioLegend). MPO concentrations were determined using a mouse specific MPO ELISA (Biolegend) according to the manufacturer's protocol on homogenized kidney samples. Blood Urea Nitrogen (BUN) levels were determined using a colorimetric assay kit (Invitrogen) on plasma samples isolated from whole blood taken at end of *Candidiasis* experiment.

### Neutrophil Isolation

Blood was obtained from healthy human volunteers with approval of the Rhode Island Hospital Institutional Review Board. Blood was collected in EDTA-containing Vacutainer tubes (BD Biosciences, San Jose, CA) and used within 15 min of venipuncture. Histopaque-1077 (Sigma Aldrich, St. Louis, MO) was used for initial cell separation followed by sedimentation through 3% dextran (400–500 kDa molecular mass, Sigma Aldrich). Contaminating erythrocytes were removed by hypotonic lysis, yielding a 95% pure neutrophil preparation of 90% viability by trypan blue dye exclusion. Neutrophils were suspended in HBSS (without Ca^2+^/Mg^2+^, Invitrogen) and placed on ice until use within 3 h.

### Neutrophil Cluster Assay

Six-well tissue culture plates (USA Scientific, Ocala, FL) were coated overnight at 4°C with Fibronectin (Fn) at a concentration of 6 μg/mL in Dulbecco's PBS (Invitrogen) and/or 1 mg/mL β-glucan (Biothera, Eagan MN). Plates were moved to 37°C for 1 h, washed twice with PBS, and air-dried. Neutrophils were resuspended to a concentration of 4 × 10^6^ cells/well in L-15 medium supplemented with 2 mg/mL glucose and 2 mL was added to each well. Cells were pretreated on ice with 10^−9^ M fMLP and 1 μM Mn^2+^ (Sigma Aldrich) for 15 min before plating. Cells were exposed to either Fn alone or Fn + β-glucan for 30 min at 37°C with 5% CO_2_, then imaged and scored for cluster formation. Dectin-1 blocking antibody (clone GE2, low-azide, Abcam, Cambridge MA) was added at 5 μg/ml, 15 mins prior to plating neutrophils.

### Quantification of Neutrophil Cluster Formation

Cluster formation was quantified using custom MatLab software. Multiple images were taken per well and clusters of neutrophils were identified by eye as regions of interconnected or overlapping cells contained within a field of view (most fields of view were 410 × 410 μm). Only cluster regions with areas 400 μm^2^ were considered, approximately the size of four tiled, non-overlapping neutrophils. To maximize consistency, a single person was used to identify every cluster region in each field of view captured. To minimize human bias, experimental conditions associated with each field of view were coded and the person doing the analysis was blinded. Fields of view were numerically characterized by the number of clusters per square millimeter and the average area of each cluster in square micrometers. Data sets from each well were averaged and all wells for each condition were ensemble averaged and plotted as average clusters per millimeter (x-axis) vs. average cluster area in square micrometer (y-axis) per condition. At least five images per well, three wells per condition each day were analyzed from at least five independent blood donors.

### Hyphal Generation and Clustering Assay

Blastoconidia of *C. albicans* were isolated from culture grown in YEPD media overnight at 37°C with shaking, washed twice in sterile water and resuspended in sterile water and counted using a hemocytometer. Hyphae differentiation was induced by plating in a 6-well plate, 2 mL/well containing 2 × 10^5^ blastoconidia in Medium 199 (Invitrogen, Carlsbad, CA) supplemented with 6 μg/mL Fibronectin (Fn, ThermoFisher, Waltham, MA) and incubating at 37°C with 5% CO_2_ for >3 h. Neutrophils (4 × 10^6^ cells/well in L-15 medium supplemented with 2 mg/mL glucose) were added to each well after being pretreated on ice with 10^−9^ M fMLP and 1 μM Mn^2+^ (Sigma Aldrich) for 15 min before plating onto hyphae with either 5 mM Chitobiose, 25 μM Bisdionin C, or uninhibited. Micrographs were taken after 30 min and clustering was assayed for cluster quantification described above.

### MTT Assay

Neutrophils were added to hyphae (as stated above) and incubated for 1 h at 37°C with 5% CO_2_ with chitobiose (5 mM, Omicron Biochemicals, South Bend, IN) or bisdionin C (25 μM, Sigma Aldrich). Neutrophils were removed by hypotonic lysis with sterile water then washed with indicator-deficient RPMI 1,640 (Invitrogen), then MTT (5 mg/mL, Tocris, Minneapolis, MN) dissolved in M199 media was added to each well and incubated for 1 h at 37°C with 5% CO_2_. Hyphae were washed twice (as above) and formazan was dissolved in DMSO (Sigma Aldrich) and transferred to a 96 well-plate and assayed at 540 nm using a Microplate BIO Kinetics Reader (BIOTEK Instruments, Winooski, VT), running DeltaSoft3 software. Data was obtained from independent experiments representing at least five independent donors.

### Flow Cytometry

Neutrophils were blocked in ice cold DPBS containing Fc receptor block (BioLegend, San Diego, CA) for 15 min on ice. Cells were treated with chitobiose (5 mM) or β-glucan (3 mg/mL) for 5 min then stained with 5 μg/mL FITC-labeled mAb for 10 min on ice. Cells were then washed twice resuspended in ice cold 10% buffered formaldehyde. Analysis was performed on a MACSQuant flow cytometer (Miltenyi Biotec, Bergisch Gladbach, Germany) and analyzed using FlowJo software (Tree Star, Ashland, OR) and gated on neutrophils.

### Chitotriosidase Activity

Candida blastoconidia (1 × 10^4^) were plated into 96 well-plate with 6 μg/ml fibronectin for 3 h in M199 media to differentiate into hyphae as above. Media was removed and neutrophils (L15 with fMLP and Mn^2+^ as above, 1 × 10^5^ per well) were added to each well along with 25 μM 4-Methylumbelliferyl β-D-N,N′-diacetylchitobioside (4-MU-DAC) in all wells (Sigma). 25 μM Bisdionin C or DMSO vehicle was added to some wells to inhibit Chit1 activity. Chitotriosidase enzyme activity releases free 4-MU which was detected using a BioTek Fluorescent plate reader (Ex340, Em460) while incubated at 37°C for 3 h. Treatment groups were conducted without 4-MU-DAC to determine absence of inherent fluorescent activity. To determine the chitotriosidase activity contained in the blood, plasma isolated from experimental animals was determined by 4-MU-DAC hydrolysis. Plasma was added with 25 μM 4-MU-DAC and incubated for 1 h at 37°C. Fluorescence readings at 1 h were obtained displayed as normalized by the subtraction of fluorescence at time 0.

### Statistics

All experimental data were analyzed using unpaired *t*-test except for CFU data which was analyzed using a negative bionomial model with classic sandwich estimation with Holm adjustment. Also, due to high variability of the magnitude of increased cytokine levels from chitobiose-treated mice compared to lactose treatment we used Pearson's correlation to determine cytokine level significance correlated to CFU in the kidney. All data presented as mean values ± SEM. Statistical significance was defined as *P* < 0.05.

## Results

### Chitotriosidase Is Released From Neutrophils in Response to Candida Hyphae

**Figure 1:**
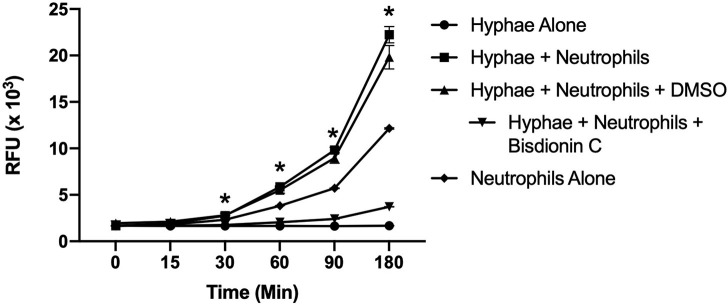
Chitotriosidase activity can be detected from neutrophils:hyphae interaction. Time course of Chit1 activity measured by the hydrolysis of 4-Methylumbelliferyl β-D-N,N′-diacetylchitobioside (4-MU-DAC) releasing fluorescent 4-MU. Neutrophils added to hyphae show significant Chit1 activity by 30 min (square, *P < 0.05 neutrophil + hyphae vs. hyphae alone), while treatment with 25 μM Bisdionin-C (downward triangle) diminishes Chit1 activity to hyphae alone levels (circle). Graph show representative example of experiment reproduced using three independent donors (*n* = 3).

Since it was determined that leukocytes, including neutrophils and macrophages, are the major source of chitotriosidase (Chit1) in mammals (14), we determined Chit1 release and activity from neutrophils in defense against Candida hyphae. To achieve this, we co-cultured normal freshly isolated neutrophils onto Candida hyphae in the presence of the extracellular matrix protein fibronectin and the fluorescent Chit1 specific substrate 4-Methylumbelliferyl β-D-N,N′-diacetylchitobioside (4-MU-DAC). We show significant increase in Chit1 activity from neutrophils and hyphae over either hyphae or neutrophils alone after 30 min (Figure 1 2,758 ± 59.8, vs. 1,659 ± 21.9, or 2,319 ± 13.4, *P* < 0.05) increasing through 180 min (22,237 ± 1,503, vs. 1,674 ± 32, or 12,153 ± 125). Also, we show the Chit1 inhibitor Bisdionin C significantly reduces Chit1 activity at 25 μM with no significant decrease in neutrophil activity from DMSO vehicle (Figure 1). Furthermore, we show no significant digestion of fluorescent substrate from hyphae alone (Figure 1) and no fluorescent activity without the addition of 4-MU-DAC in all groups (Supplementary Figure 1).

### Chitotriosidase Is Not Essential in Defense Against Systemic Candidiasis

**Figure 2:**
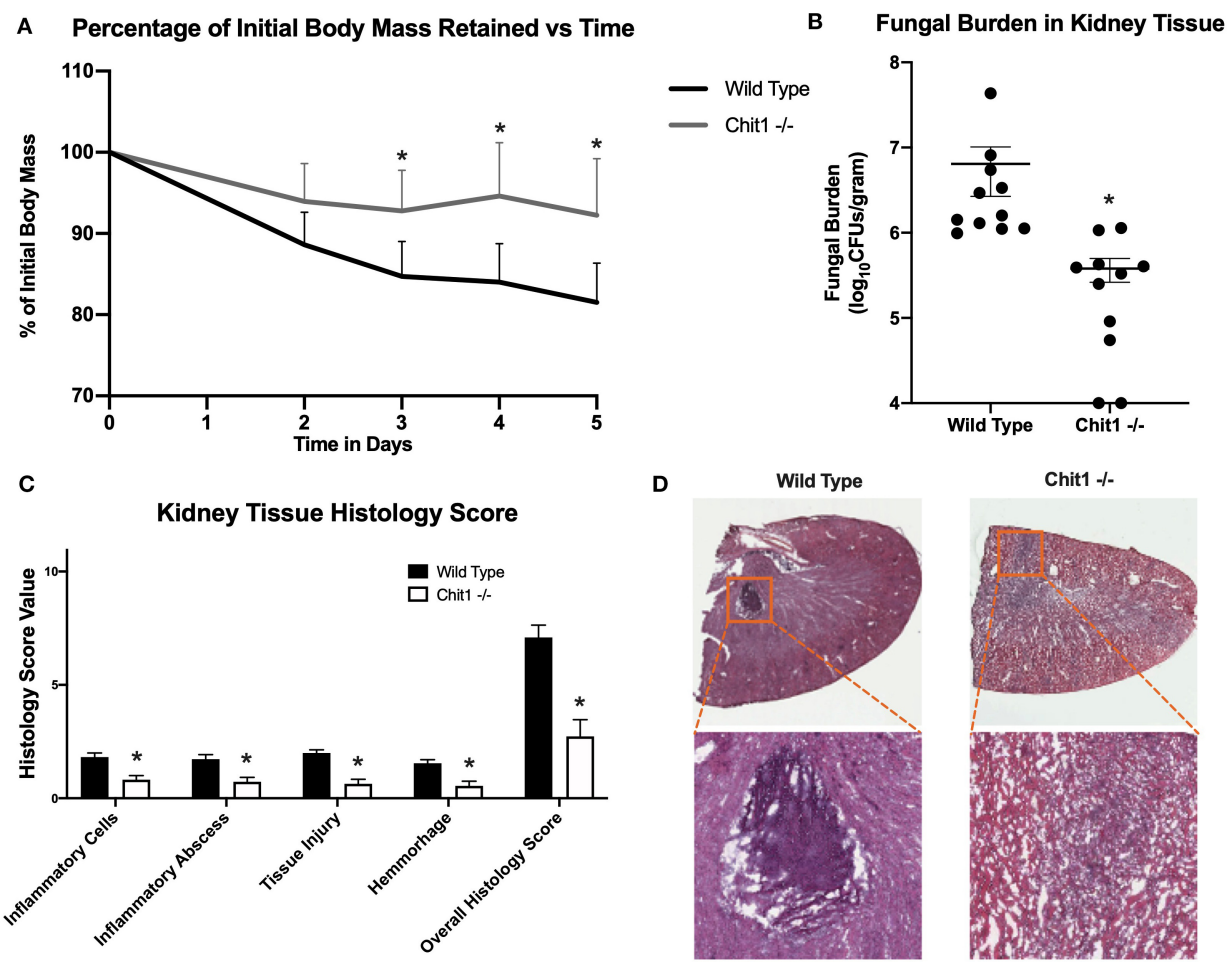
Chitotriosidase deficiency results in a protective phenotype against the systemic candidiasis compared to wild type mice. **(A)** Chit1^−/−^ mice show a significant reduction of infection-induced weight loss compared to that observed in wild type mice on days 3, 4, and 5 post infection (*P < 0.05 Chit1^−^/^−^ vs. WT). **(B)** Chit1^−/−^ mice show significantly less Candida dissemination in the kidney tissue 5 days after systemic infection compared to wild type (*P < 0.05 Chit1^−/−^ vs. WT, *n* = 11 mice/group). **(C)** Histology scores for individual categories as well as the overall histology scores are displayed showing decreased levels in Chit1^−/−^ mice compared to WT mice 5 days after systemic infection (*P < 0.05 Chit1^−/−^ vs. WT, *n* = 11 mice/group). **(D)** H&E stain of the fungally-infected kidney 5 days post infection demonstrating marked inflammation with the formation of abscesses shown in enlargement. Chit1^−/−^ mice show decreased inflammation and abscess formation compared to WT mice.

**Figure 3:**
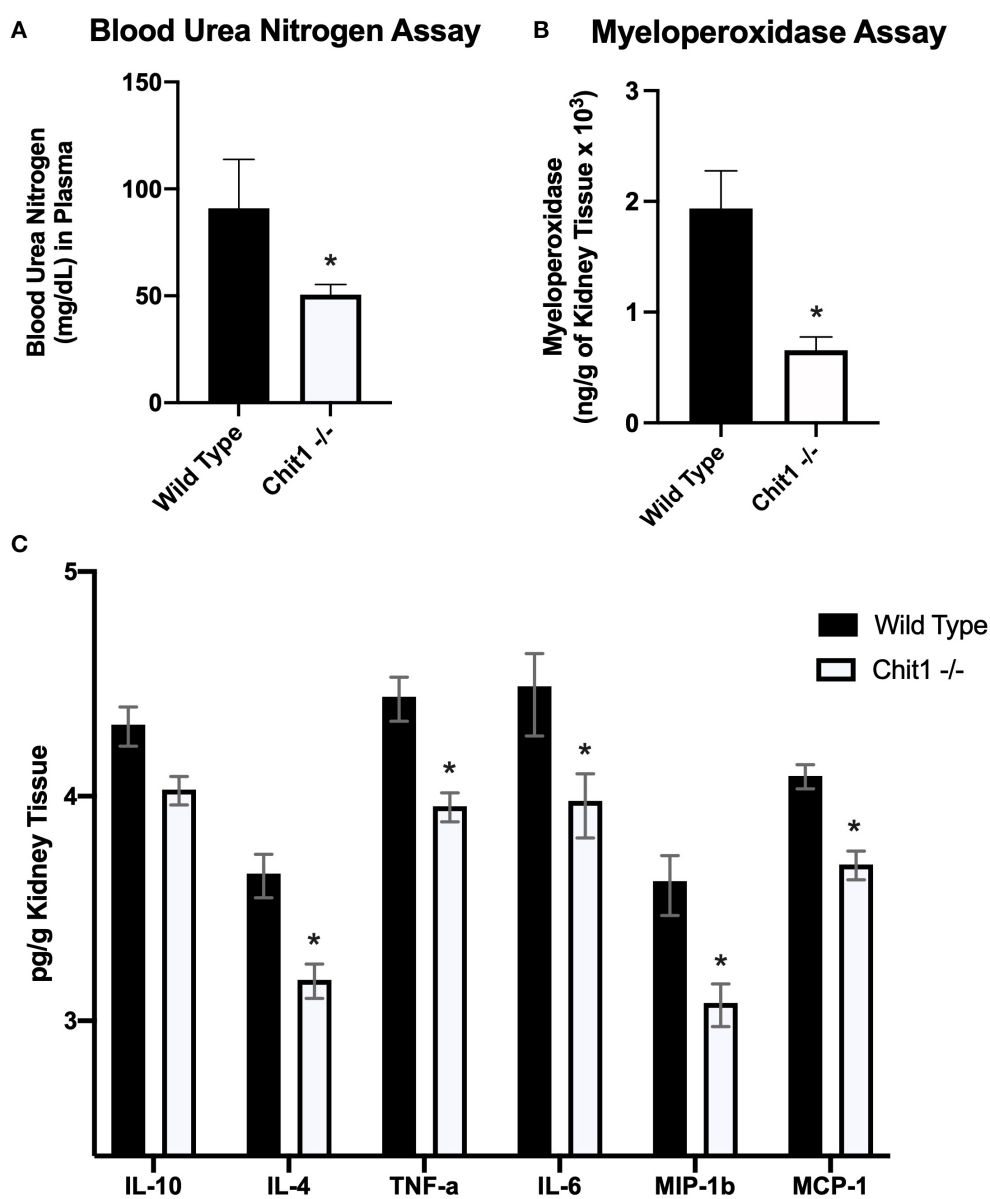
Chitotriosidase deficiency results in a protective phenotype against the systemic candidiasis compared to wild type mice 5 days after infection. **(A)** Urea nitrogen levels in the blood was significantly different between Chit1^−/−^ and WT mice (*P < 0.05 Chit1^−/−^ vs. WT, *n* = 11 mice/group). **(B)** Myeloperoxidase levels in the kidney was significantly different between Chit1^−/−^ and WT mice (*P < 0.05 Chit1^−/−^ vs. WT, *n* = 11 mice/group). **(C)** Pro-inflammatory and chemotactic cytokine levels of the kidney are significantly decreased in Chit1^−/−^ compared to WT mice (*P < 0.05 Chit1^−/−^ vs. WT, *n* = 11 mice/group, IL-10 not significant).

**Figure 4:**
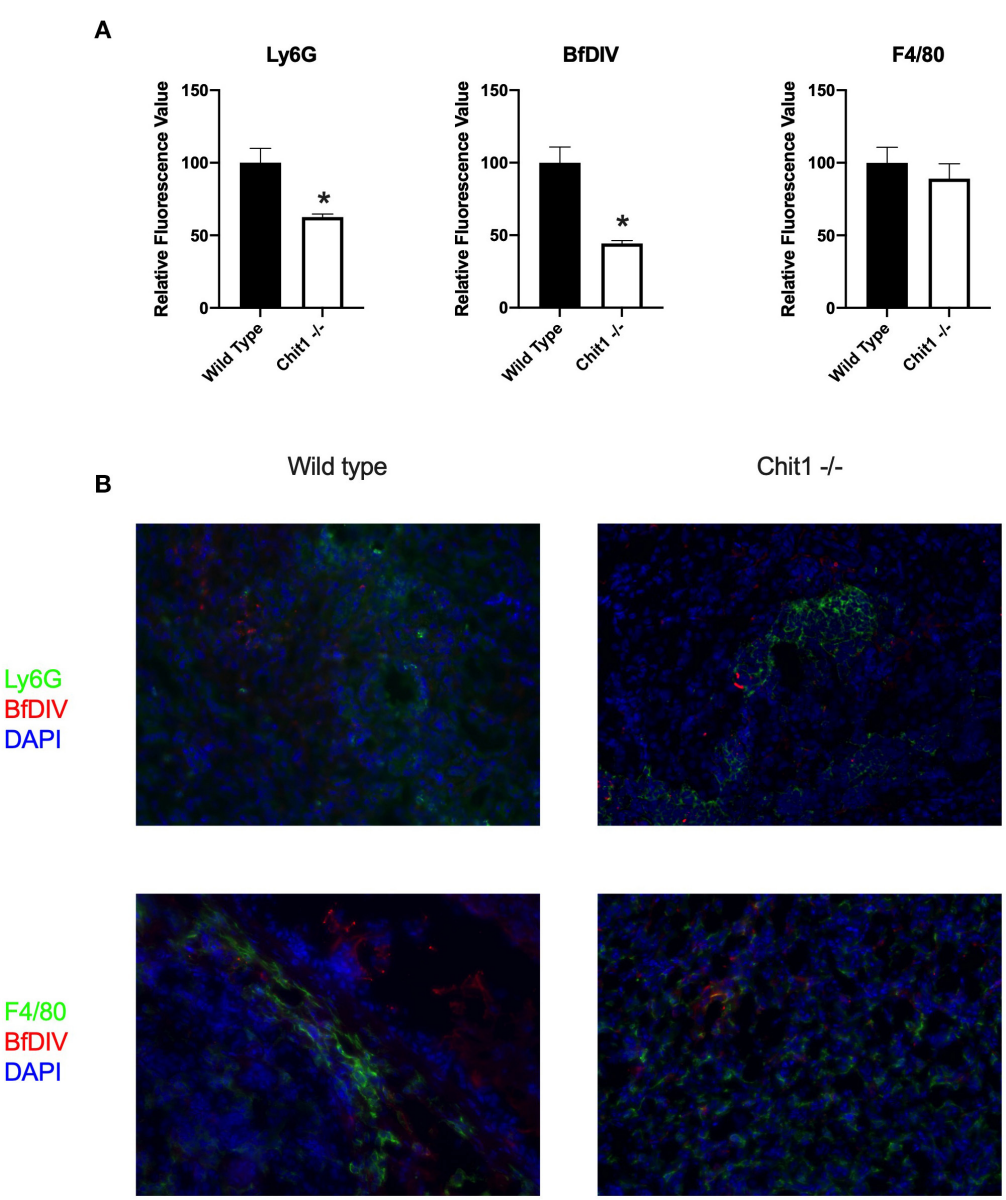
Chitotriosidase deficiency results in a decreased neutrophils and Candida but not macrophages in the kidney during systemic candidiasis compared to wild type mice 5 days after infection. **(A)** Using quantitative immunofluorohistochemistry, Chit1^−/−^ mice show significantly decreased levels of neutrophils (Ly6G) and yeast (BfDIV) but not macrophages (F4/80) compared to WT mice (*P < 0.05 Chit1^−/−^ vs. WT, *n* = 11 mice/group). **(B)** Immunofluorohistochemistry of the fungally-infected kidney demonstrating decreased neutrophil and yeast detection with no difference in macrophage detection in the Chit1^−/−^ mice compared to wild type.

To determine the importance of Chit1 in the inflammatory response to fungal infection we used a mouse model of systemic candidiasis. Infection was established by injecting chitotriosidase knock out (Chit1^−/−^) and sex and age-matched wild type (WT) mice with 1 × 10^5^ blastospores of *C. albicans* strain SC5314 via the tail vein. At day 5 (the experimental endpoint due to 20% weight loss in WT mice) after infection with *C. albicans*, Chit1^−/−^ mice showed minimal weight loss by day 5 post infection compared to WT (Figure 2A), 92.3 ± 6.9 vs. 81.5 ± 4.8 %, *P* < 0.05). The fungal burden in the kidney was significantly lower in the Chit1^−/−^ mice at day 5 post infection compared to WT (Figure 2B), 5.5 ± 1.0 vs. 6.8 ± 0.2 log_10_ CFUs/gram kidney tissue, *P* < 0.05). Additionally, histological analysis of the H&E stained kidney (Figure 2D) showed significant decreases in inflammatory scores in Chit1^−/−^ compared to WT mice (Figure 2C). Likewise, kidney function measured by blood urea nitrogen levels showed significantly decreased levels in Chit1^−/−^ compared to WT mice (Figure 3A). Moreover, leukocyte response in the kidney measured by MPO showed significantly decreased levels in Chit1^−/−^ compared to WT mice (Figure 3B). Also, inflammatory cytokine analysis of kidney tissue showed significant decrease in cytokine and chemokines in Chit1^−/−^ compared to WT mice (Figure 3C). Furthermore, extending this trend, immunohistochemical analysis using antibodies against neutrophils (Ly6G), macrophages (F4/80), and yeast (BfDIV) show significantly decreased detection of neutrophils and yeast in kidney tissues of Chit1^−/−^ compared to wild type mice (Figures 4A,B) No significant difference was determined in macrophage levels (Figures 4A,B). Taken together, these data indicate the loss of Chit1 is beneficial in mounting an efficient systemic response against systemic candidiasis in a mouse model.

### Chitriosidase Endproduct Chitobiose Attenuates Neutrophil Aggregation

**Figure 5:**
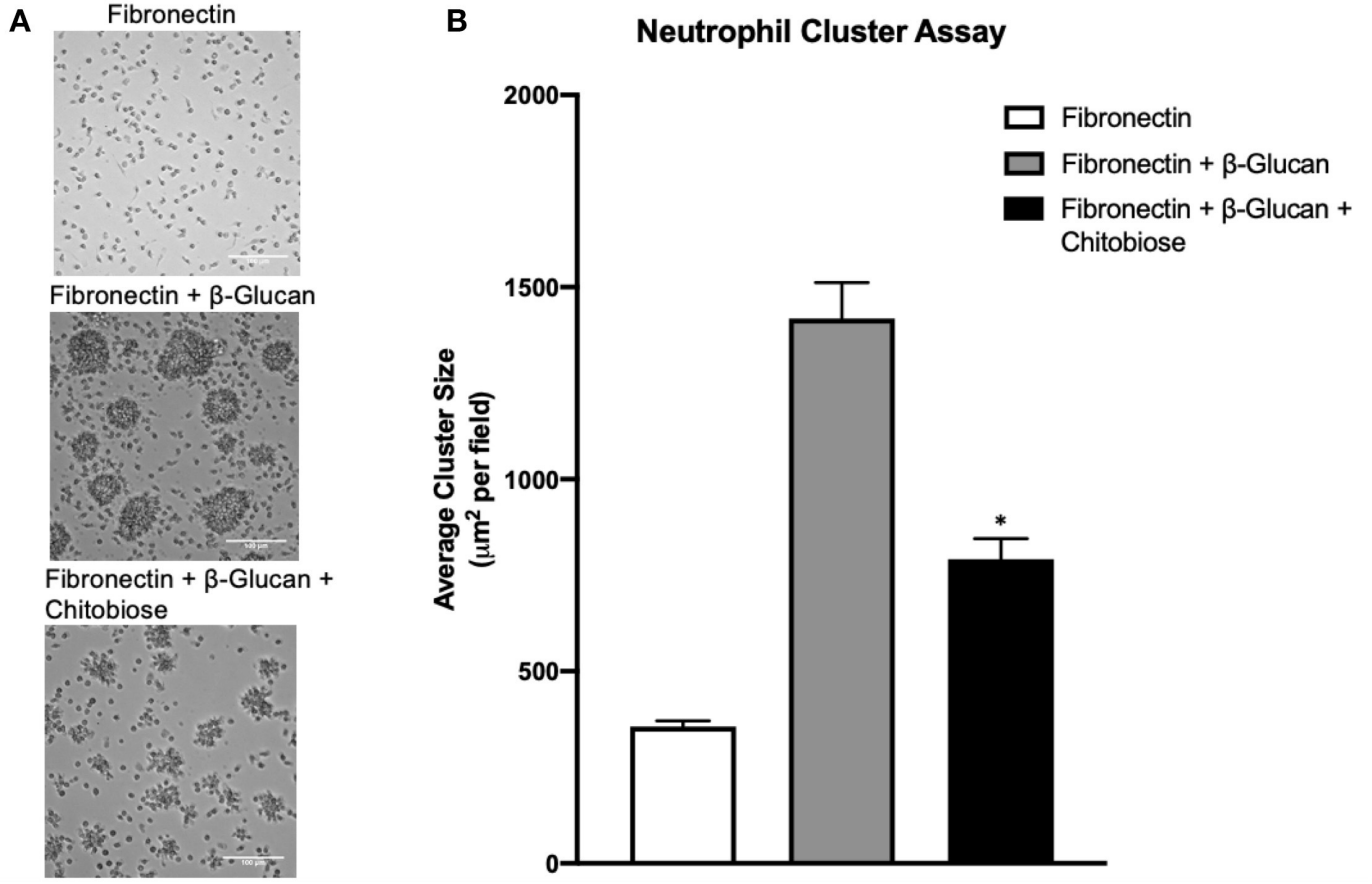
Chitobiose inhibits neutrophil clustering *in vitro*. Using a reductionist model of fungal hyphae, primed neutrophils showed decreased clustering/swarming in the presence of chitobiose. **(A)** Micrographs show neutrophil aggregation 30 min after they were added to plates pre-coated with either Fn of Fn + β-glucan with or without chitobiose (all images were taken at 20x magnification). **(B)** Cluster formation was quantified using a custom MatLab script to quantify the average area of neutrophil clusters in μm^2^ for each treatment group (the Average ± SEM is also recorded, *P < 0.05 FB vs. FBC, *n* = 5 independent experiments/blood donors).

To model neutrophil recognition of fungal hyphae, which are too large for internalization, we immobilized soluble β-glucan onto a tissue culture surface as used previously in our laboratory (12, 24). Since neutrophil interactions with fungal hyphae occur within the infected tissue in the presence of extracellular matrix, we added the ubiquitous tissue matrix component, fibronectin. Additionally, since neutrophils reach a primed state during diapedesis into the tissue, we used a sub-activation concentration of fMLP (10^−9^ M) to achieve a primed state in our *in vitro* assay. Homotypic aggregation of neutrophils was observed in the presence of both Fn and β-glucan within 30 min, but not in the presence Fn alone [as described previously (24), representative examples shown in Figure 5A]. Considering that the degradation process of the chitin polysaccharide primarily results in the release of chitobiose, we added 5 mM chitobiose (dose curve shown in Supplementary Figure 3) and neutrophils concurrently onto tissue culture surfaces that had been previously coated with both immobilized β-glucan and Fn as described above. As shown in Figure 5B, the presence of chitobiose resulted in a significant decrease in cluster size compared to Fn + β-glucan (791 ± 120 vs. 1,418 ± 208 μm^2^, *P* < 0.05). However, chitobiose treatment did not result in a significant change in number of clusters observed when compared to the Fn + β-Glu treatment (Data not shown). These data show we were able to recreate the functional inhibition of chitobiose on CR3-mediated neutrophil migration in our *in vitro* reductionist fungal hyphae model system. We next determined the effect of chitobiose on neutrophil clustering against Candida hyphae.

### Chitobiose Decreases Neutrophil Aggregation on *Candida* Hyphae

**Figure 6:**
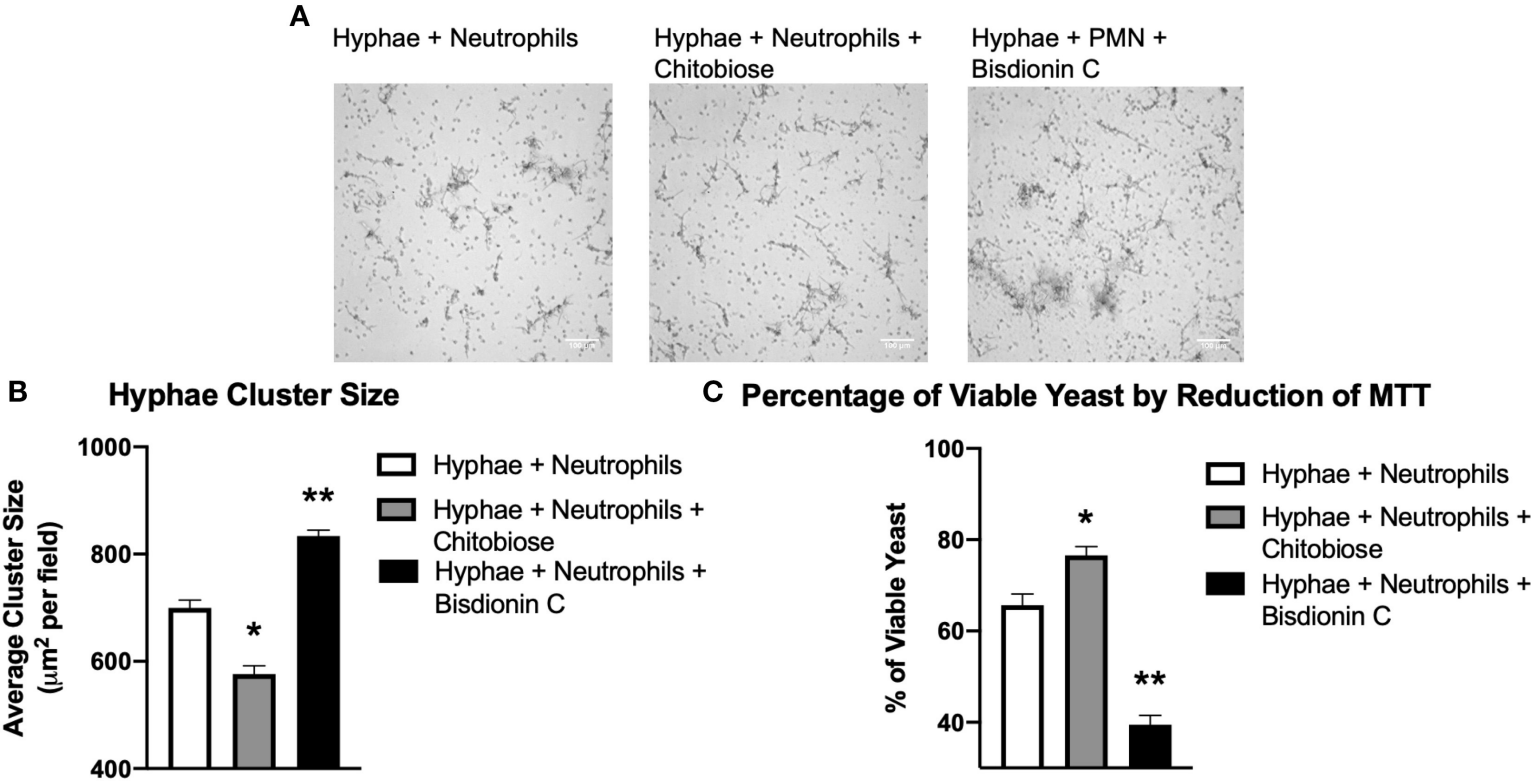
Chitobiose inhibits neutrophil clustering to and killing of *Candida* hyphae however both are enhanced by Chit1 activity inhibition. Primed neutrophils plated on tissue culture surfaces in the presence of Candida hyphae showed decreased clustering/swarming in the presence of chitobiose. **(A)** Micrographs show neutrophil aggregation 30 min after they were plated on tissue culture surfaces in the presence of *Candida* hyphae and treated with either chitobiose (5 mM) or bisdionin C (25 μM). All images were taken at 10x magnification. **(B)** Chitobiose treatment resulted in decreased average cluster size compared to untreated, while treatment with Bisdionin C showed a significant increase in cluster size. No significant difference was observed in the number of resulting clusters between the chitobiose and Bisdionin C treatments. **(C)** Chitobiose treatment resulted in a significant increase in the percentage of viable Candida observed while the Bisdionin C resulted in a significant decrease in the percentage of viable *Candida*. Cluster formation was quantified using custom MatLab script and graphed as cluster area in μm^2^ per condition. Average±SEM, *P < 0.05 chitobiose vs. untreated, **P < 0.05 Bisdionin C vs. untreated, *n* = 5 independent experiments/blood donors.

To determine the effect of excess chitobiose on the neutrophil response to *Candida* hyphae, we grew *C. albicans* hyphae in tissue culture wells containing Fn. Neutrophil clustering around fungal hyphae structures (Figure 6A) were observed to be recapitulating the neutrophil aggregation seen in Fn+β-glucan-coated tissue culture surfaces. Furthermore, chitobiose treatment of neutrophils exposed to fungal hyphae resulted in a modest, but significant, decrease in neutrophil cluster size when compared to untreated neutrophils (Figure 6B), 576.2 ± 35.1 vs. 699.6 ± 32.9 μm^2^, *P* < 0.05). These findings proved to be consistent with the results observed when applying the chitobiose treatment to neutrophils added to tissue culture surfaces coated with immobilized β-glucan. Furthermore, treatment with the chitotriosidase inhibitor, Bisdionin-C (25 μM), results in an increase in neutrophil clustering size around Candida hyphae compared to untreated neutrophils (Figure 6B), 833.6 ± 24.9 vs. 699.6 ± 32.9 μm^2^, *P* < 0.05).

### Chitobiose Decreases Neutrophil Killing of *Candida* Hyphae

Since chitobiose treatment demonstrated a diminishing effectiveness of neutrophil clustering around Candida hyphae in Figure 6, we determined the effect of chitobiose on the efficiency of hyphal killing using MTT to assay fungal viability. *Candida* hyphae were grown (as described above) in the presence of Fn and neutrophils were added with or without the presence of 5 mM chitobiose. As shown in Figure 6C, chitobiose treatment resulted in a decrease in neutrophil killing resulting in a significant increase in viability compared to untreated neutrophils (76.6 ± 4.4 vs. 65.7 ± 5.5 % viability, *P* < 0.05). Furthermore, Bisdionin-C treatment (25 μM) significantly enhanced neutrophil killing of Candida hyphae compared to untreated neutrophils (39.5 ± 4.6 vs. 65.7 ± 5.5 % viability, *P* < 0.05). Taken together, the *in vitro* findings strongly identify chitobiose directly interferes with neutrophil inflammatory responses resulting in decreased clustering and subsequent killing to *Candida* hyphae.

### Chitobiose-Mediated Neutrophil Inhibition Is Linked to Lectin-Like Site on CR3

**Figure 7:**
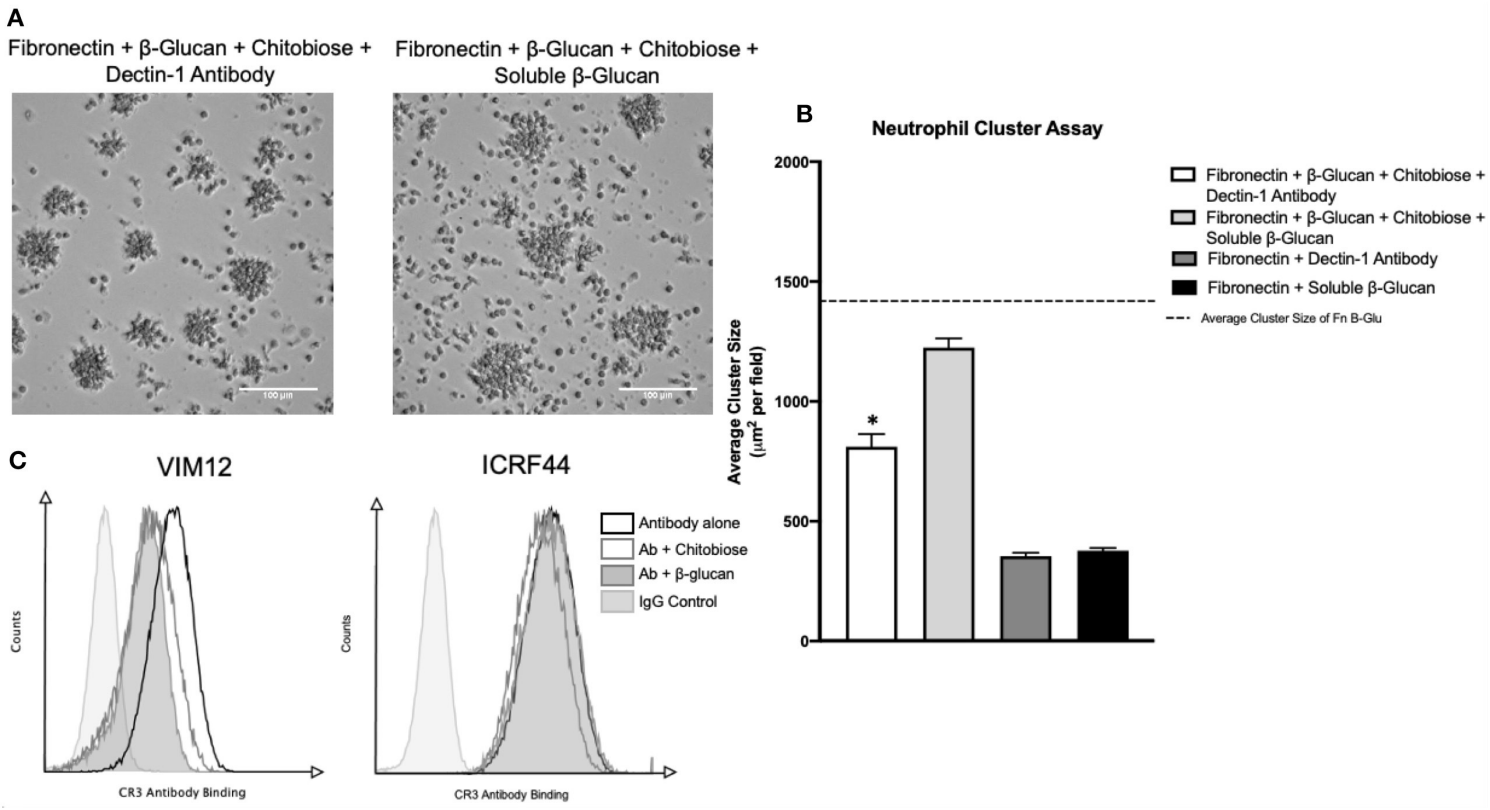
Chitobiose inhibits neutrophil function by interaction at CR3 lectin-like site. **(A)** Micrographs show neutrophil aggregation 30 min after they were added to plates pre-coated with either Fn of Fn + β-glucan and treated with chitobiose as well as either soluble β-glucan or Dectin-1 blocking antibody (all images were taken at 20x magnification). **(B)** Addition of soluble β-glucan not blocking Dectin-1 prevents chitobiose-reduced neutrophil clustering. No difference in cluster number by either treatment was observed, showing clustering of Dectin-1 treatment with chitobiose was still significantly decreased compared to fn+β-glucan alone (dotted line). Cluster formation was quantified using custom MatLab script and graphed as cluster area in μm^2^ per condition. Average ± SEM, *P < 0.05 Dectin-1 + chitobiose vs. untreated, *n* = 5 independent experiments/blood donors. **(C)** Flow cytometry was performed and is presented as CR3 Antibody Binding (x-axis) vs. Counts (y-axis). Groups shown as CR3-antibody alone (black line, no fill), Ab with chitobiose (gray line, no fill), Ab with β-glucan (gray line, gray fill), IgG control (light gray line, light gray fill) *n* = 3 independent experiments/blood donors.

Recognition of *Candida* is achieved by the binding of the cell wall β-glucan to either the integrin CR3 or by the C-type lectin Dectin-1 located on the neutrophil surface. Prior work showed the monosaccharide comprising chitobiose (N-acetyl-D-glucosamine, NADG) can inhibit β-glucan binding to the lectin-site of the integrin CR3 (CD11b/CD18). We therefore determined if the mechanism of neutrophil inhibition by chitobiose is the result of β-glucan recognition interference. To accomplish this, we added either Dectin-1 blocking antibody or excess soluble β-glucan (Figure 7A) to the immobilized β-glucan clustering assay. Results show the addition of soluble β-glucan (960 μg/ml, dose curve shown in Supplementary Figures) to chitobiose-treated Fn+β-glucan clustering assay significantly blocked the chitobiose induced inhibition (Figure 7B), 1,206 ± 96 vs. 885 ± 108 μm^2^, *P* < 0.05, soluble-β-glucan vs. untreated), while Dectin-1 blocking antibody (5 μg/ml) has no significant effect. Furthermore, the addition of both soluble β-glucan and dectin-1 antibody has no significant increase on cluster number (Data not shown). To further link chitobiose effect to the lectin-like site, we determined if chitobiose could reduce the binding of lectin-like site specific CR3 antibody (VIM12) compared to an I-domain specific antibody (ICRF44). Results show that chitobiose treated neutrophils (5 mM) had decreased VIM12 binding compared to untreated neutrophils, while having no effect on ICRF44 binding (Figure 7C). Soluble β-glucan (960 μg/ml) was used as a positive control and showed similar binding effects as chitobiose.

### Chitobiose Treatment Enhances Severity of Systemic Candidiasis *in vivo*

**Figure 8:**
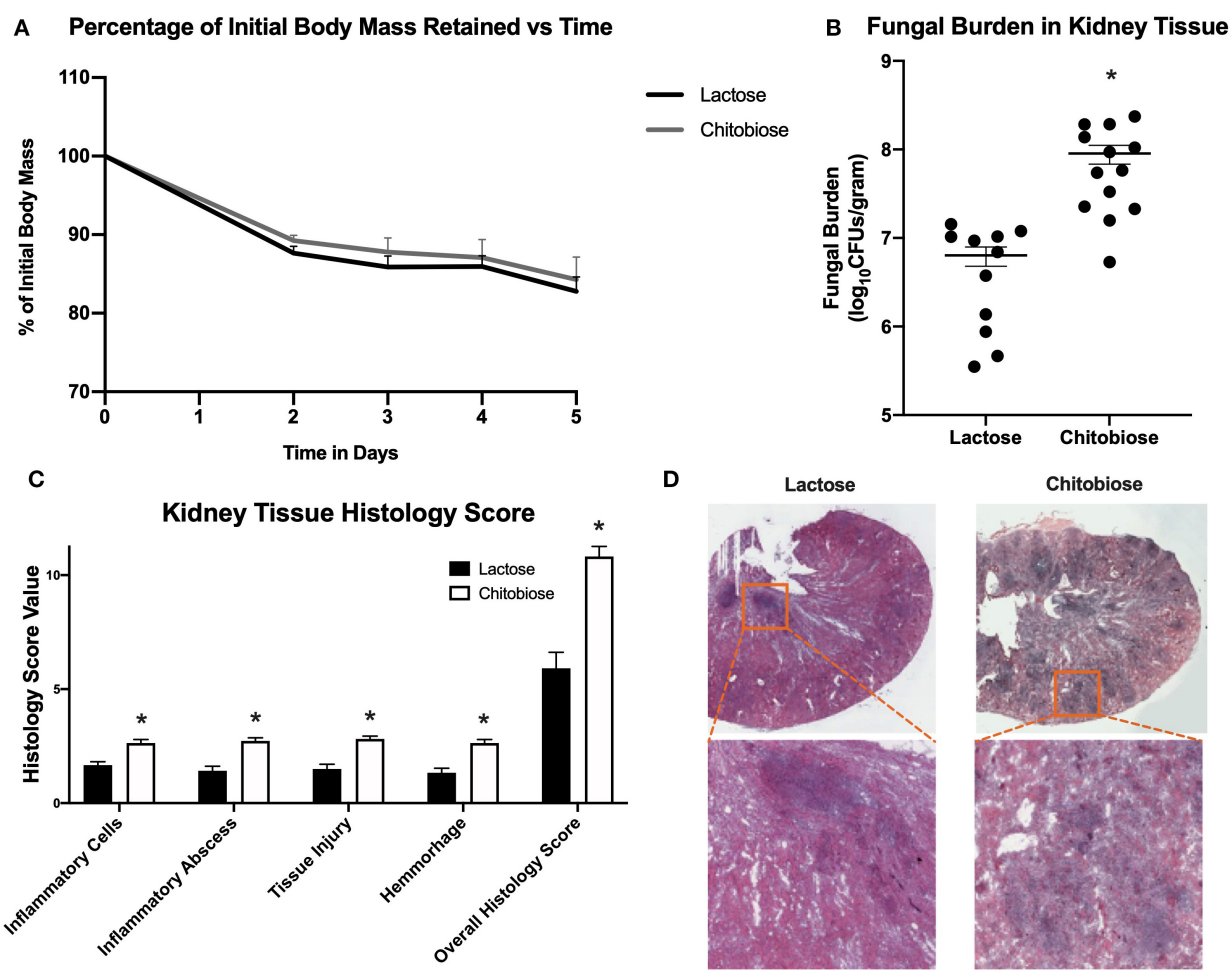
Systemic treatment of chitobiose exacerbates candidiasis. **(A)** Chitobiose treatment (100 mg/kg) results in no significant reduction of infection-induced weight loss compared to lactose-treated. **(B)** However, chitobiose treated mice show significantly more Candida dissemination in the kidney tissue 5 days after systemic infection compared to lactose-treated mice (*P < 0.05 chitobiose vs. lactose, *n* = 11 mice/ group). **(C)** Histology scores for individual categories as well as the overall histology scores are displayed showing significantly increased levels in chitobiose-treated mice compared to lactose-treated mice 5 days post infection (*P < 0.05 chitobiose vs. lactose, *n* = 11 mice/group). **(D)** H&E stain of the fungally-infected kidney 5 days post infection demonstrating marked inflammation with the formation of abscesses shown in enlargement. Chitobiose mice show increased inflammation and abscess formation compared to lactose-treated mice.

**Figure 9:**
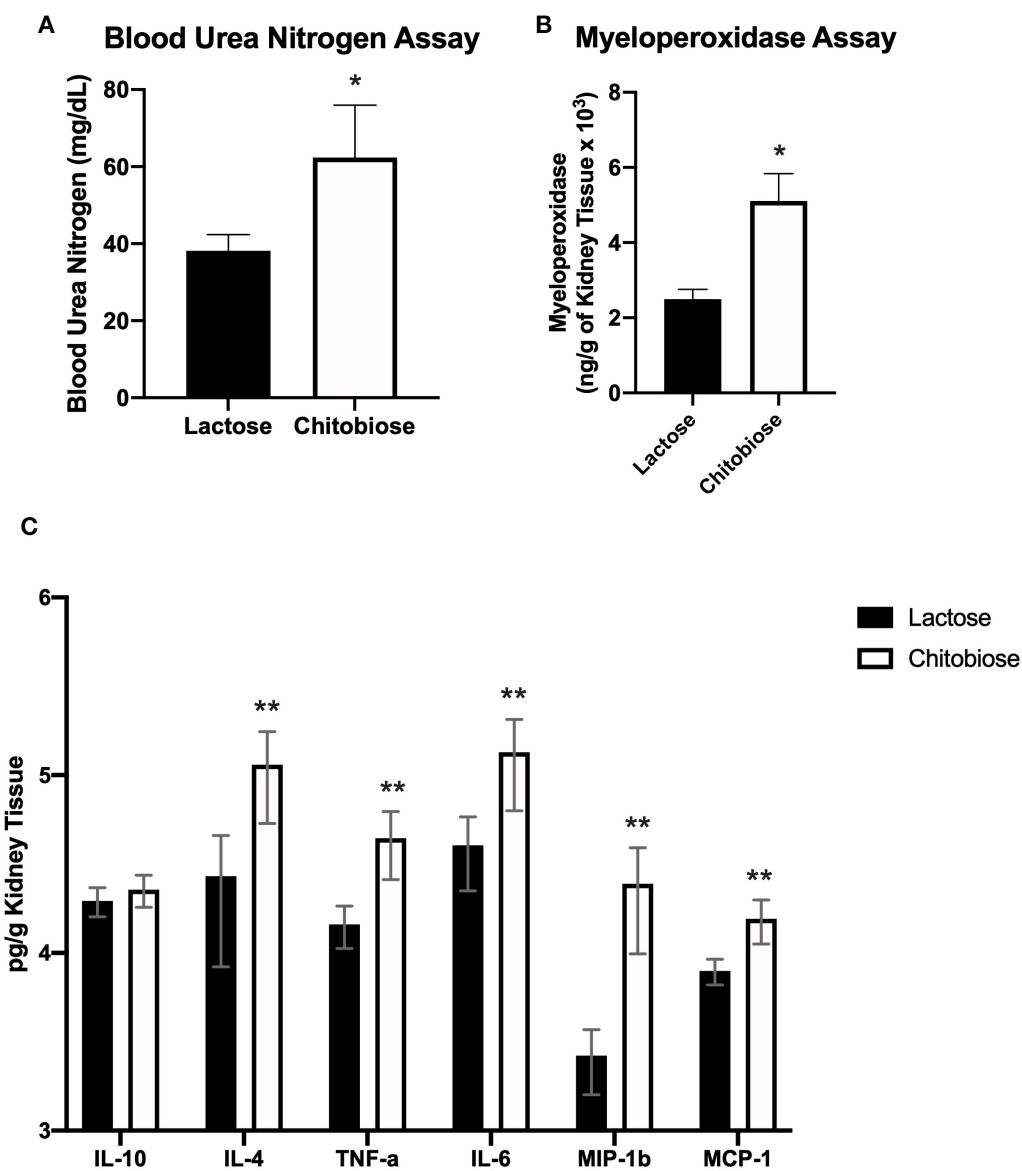
Systemic treatment of chitobiose exacerbates candidiasis measured 5 days after infection. **(A)** Urea nitrogen levels in the plasma were significantly increased in chitobiose compared to lactose-treated mice (*P < 0.05 chitobiose vs. lactose, *n* = 11 mice/group). **(B)** Myeloperoxidase levels in the kidney were significantly increased in chitobiose-treated compared to lactose-treated mice (*P < 0.05 chitobiose vs. lactose, *n* = 11 mice/ group). **(C)** Pearson correlation Pro-inflammatory and chemotactic cytokine levels to CFU's of the kidney are significantly increased in chitobiose compared to lactose-treated mice (**P < 0.05 chitobiose vs. lactose-treated, *n* = 11 mice/group, IL-10 not significant).

**Figure 10:**
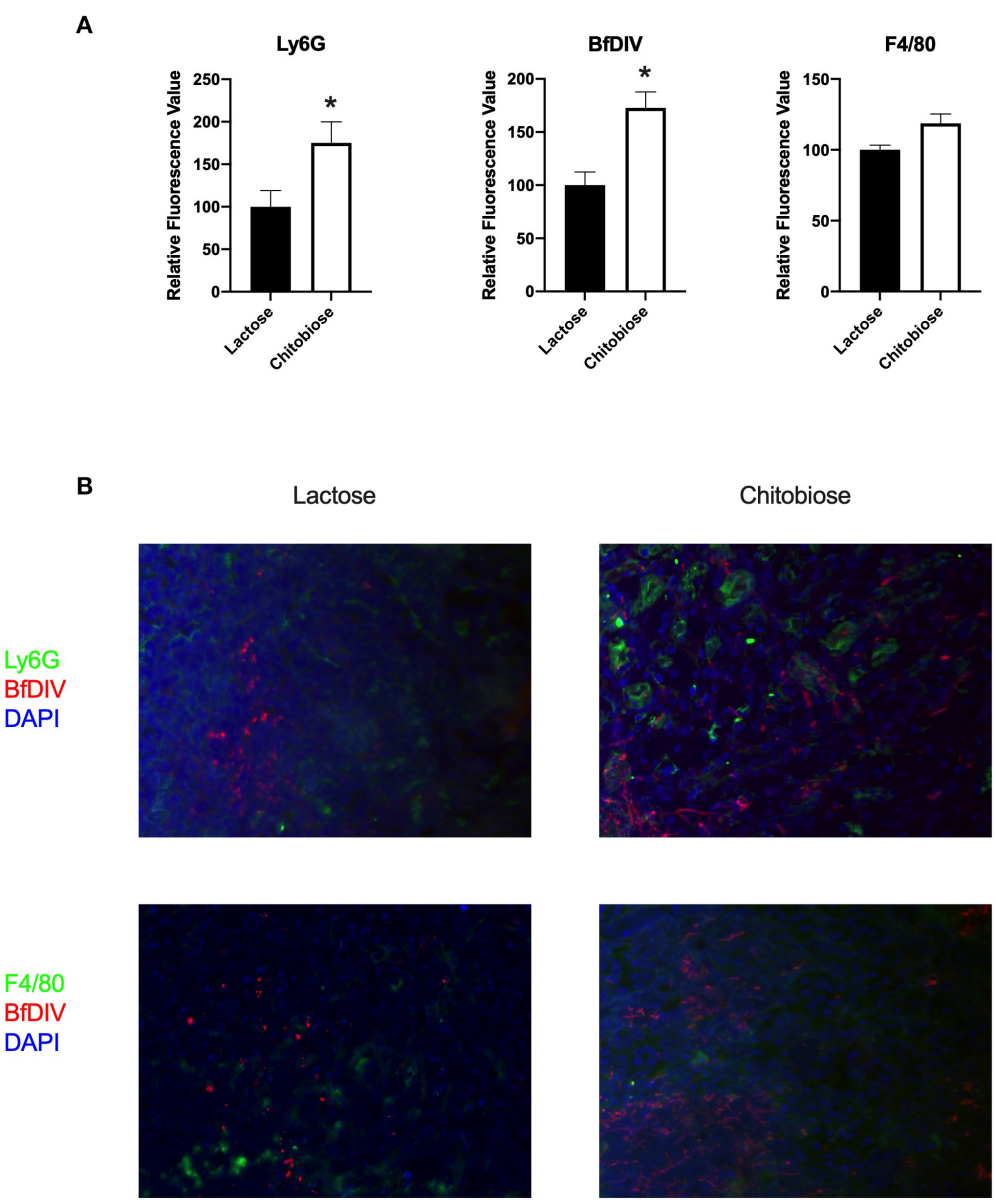
Systemic treatment of chitobiose results in increased neutrophils and Candida but not macrophages in the kidney during systemic candidiasis compared to lactose-treated mice 5 days after infection. **(A)** Using quantitative immunofluorohistochemistry, chitobiose-treated mice show significantly increased levels of neutrophils (Ly6G) and yeast (BfDIV) but not macrophages (F4/80) compared to lactose-treated mice (*P < 0.05 chitobiose- vs. lactose-treated, *n* = 11 mice/group). **(B)** Immunofluorohistochemistry of the fungally-infected kidney demonstrating increased neutrophil and yeast detection with no difference in macrophage detection in the chitobiose-treated compared to lactose-treated mice.

Since chitobiose was able to inhibit neutrophil functions *in vitro*, we set out to determine if the addition of chitobiose resulted in a less effective anti-*Candida* neutrophil response *in vivo*. To accomplish this, we treated C57/BL6 mice with either 100 mg/kg chitobiose or lactose as a control disaccharide. At day 5 after infection with *C. albicans*, chitobiose treated mice showed no significant weight loss compared to lactose-treated mice (Figure 8A 84.3 ± 2.8 vs. 83.0 ± 1.8 % *P* > 0.05), however the fungal burden in the kidney was significantly higher in the chitobiose-treated mice compared to lactose-treated mice (Figure 8B 8.0 ± 1.5 vs. 6.8 ± 1.3 log_10_CFUs/gram, *P* < 0.05). Additionally, histological analysis of the H&E stained kidney (Figure 8D) showed significant increases in inflammatory scores in Chitobiose-treated compared to lactose-treated mice (Figure 8C). Likewise, kidney function measured by blood urea nitrogen levels showed significantly elevated levels in Chitobiose-treated compared to lactose-treated mice (Figure 9A). Moreover, leukocyte response in the kidney measured by MPO showed significantly increased levels in Chitobiose-treated compared to lactose-treated mice (Figure 9B). Additionally, inflammatory cytokine analysis of kidney tissue showed significant correlation of increased cytokine and chemokines levels compared to CFU in Chitobiose-treated compared to lactose-treated mice (Figure 9C). Furthermore, extending this trend, immunohistochemical analysis show significantly increased detection of neutrophils and yeast in kidney tissues of Chitobiose-treated compared to lactose-treated mice (Figures 10A,B) No significant difference was determined in macrophage levels (Figures 10A,B). These results indicate the addition of excess chitobiose during systemic candidiasis is detrimental to mounting an efficient systemic response resulting in a reduced clearance of Candida.

## Discussion

Although human cells do not produce chitin, they are exposed through contact with chitin containing pathogens including *Candida albicans*. There are currently two identified human chitinase proteins that are enzymatically active, acidic mammalian chitinase (AMCase) and Chit1 (15). The former has an acidic isoelectric point (pI) allowing optimal activity in the gastrointestinal tissue, where the Chit1 has a pI closer to that of inflamed tissue and is therefore the focus of this current study (25). Furthermore, Chit1 is present in primary, secondary and tertiary neutrophilic granules (26). However, we show that Chit1 is not an essential component of the inflammatory response to systemic candidiasis. Similarly, of the myriad of known neutrophil specific immunodeficiencies caused by genetic abnormalities linked to *Candida* infection, including Dectin-1, CD18, CARD9, MPO, and enzymes of the NADPH oxidase pathway (6), to date chitotriosidase has not been linked in an increase in fungal susceptibility (6, 27). In fact, our data suggest the absence of Chit1 was beneficial as the genetic deficient animals demonstrated nearly a full recovery of infection induced weight loss and decreasedfungal burden. Chit1 deficient mice show significant alterations in inflammatory leukocyte accumulation in the kidney measured by MPO and quantitative immunohistochemistry, indicating more efficient direct action of leukocytes against Candida compared to wild type mice. Therefore, interference with efficient inflammatory response could be caused by either Chit1 protein itself, or the end-product of chitin breakdown, chitobiose. For instance, we found that plasma levels of Chit1 is parallel with the extent of systemic candidiasis in experimental animals used throughout this study (Supplementary Figure 2). If the Chit1 protein is inhibiting the inflammatory response, then increased susceptibility to fungal infections would be associated with disorders overexpressing Chit1. In addition to fungal and bacterial diseases, chitotriosidase expression is increased in several non-infectious inflammatory diseases. Specifically, elevated Chit1 serum levels are associated with atherosclerosis, COPD, Alzheimer's disease and β-thalassemia, and is a biomarker for Gaucher's disease which affects lysosomal storage (17, 28–31). However, to date, no correlation has been linked between increased bacterial/fungal infections and increased chitotriosidase enzyme or polymorphism of the protein (32). Chitin makes up ~6% of the dry weight of *Candida albicans* cell wall (33). This provides for the potential to create a substantial amounts of chitobiose during hydrolysis of chitin due to leukocyte-released chitiotriosidase at the Candida:leukocyte interface. Given this possibility, we focused on chitobiose as the potential link to the obstructed immune response.

In infected tissues, *C. albicans* occurs in two morphologic forms, unicellular yeast and filamentous hyphae. The virulence associated with *C. albicans* is directly correlated with its ability to transition between the two forms (34). The unicellular yeast form is small and readily cleared by neutrophil phagocytosis. In contrast, the filamentous and highly elongated pseudo-hyphal and hyphal forms are substantially longer than the neutrophil and thus, cannot be cleared by internalization therefore, neutrophils must employ other effector mechanisms to kill hyphae, including degranulation (35). The hyphal form is a hallmark histological marker of tissues subjected to fungal based infection, where numerous neutrophils are observed to be clustered around the much larger Candida hyphae. Therefore, our lab has developed a CR3-dependent homotypic aggregation system as a reductionist model of the response of neutrophils to fungal hyphae in the absence of phagocytosis, by plating neutrophils on immobilized β-glucan and fibronectin. We utilized this model to address the ability of chitobiose to inhibit neutrophil clustering/swarming. Consequently, the addition of chitobiose was able to significantly inhibit the neutrophil clustering response. Moreover, the presence of chitobiose significantly inhibited both neutrophil clustering onto and killing of Candida hyphae. Chitotriosidase belongs to the glycosyl hydrolase 18 family of mammalian chitinase (14). Several inhibitors have been developed against this family with Bisdionin-C showing specificity toward Chit1 (36). Not only were we able to prevent the chitobiose-induced decrease in clustering to Candida hyphae, but further amplify the clustering and neutrophil induced killing in the absence of exogenous chitobiose. If preventing the creation of chitobiose by eliminating Chit1 activity, the addition of chitobiose would decrease neutrophil effectiveness *in vivo*. Chitobiose has been shown to be well-tolerated in rodents (37), and we were able to decrease the effectiveness of the inflammatory response to systemic candidiasis *in vivo*.

The cell wall of *Candida* contains a mesh of several long chain polysaccharides including β-glucan, mannan and chitin (38). These molecules are not present in mammalian cells therefore can be recognized by cells of the immune system and are referred to as pathogen-associated molecular patterns (PAMPs). CR3 is unique among integrins as it has two spatially distinct binding sites, the so-called I-domain and the lectin-like domain, that bind completely different ligands and results in differing cellular responses. In human neutrophils, CR3 has been demonstrated to be the major PAMP receptor binding to β-glucan through its lectin-like domain. Over several publications, Gordon Ross and colleagues showed the monosaccharide, NADG inhibited β-glucan binding to neutrophil CR3 (11, 39). The addition of NADG inhibited the Fc receptor mediated lysis of opsonized tumor cells by neutrophils (40). Likewise, NADG disrupted IL-2-activated human lymphocytes binding to *C. albicans* hyphae (41). These studies linked the bioactivity of NADG to the lectin-like domain of the integrin CD18/CD11b (CR3, Mac-1). Therefore, we linked chitobiose to interfering with neutrophil function through the CR3 lectin site by successfully out-competing the inhibition with the addition of soluble β-glucan. However, further evidence was necessary to link the involvement of CR3.

Antibodies against specific regions of CR3 have been used to determine which epitopes of CD11b are involved in receptor interactions. For instance, Thorton et al. used a panel of antibodies recognizing sites across CD11b and found antibodies against the lectin-like domain (including VIM12) were able to inhibit β-glucan binding (21). Utilizing the same strategy, we determined chitobiose was able to reduce the binding of VIM12 but not the antibody against the I-domain antibody ICRF44. This added to the likelihood that chitobiose is acting on neutrophil function through binding to the lectin site.

In summary, these data show that the enzymatic activity of chitinase produces a local concentration of chitin breakdown products (chitobiose) capable of inhibiting neutrophil anti-fungal functions through interactions at the lectin-like domain of CR3. Therefore, chitotriosidase inhibition can be useful as a target for anti-fungal treatment.

## Data Availability Statement

The raw data supporting the conclusions of this article will be made available by the authors, without undue reservation.

## Ethics Statement

The animal study was reviewed and approved by Rhode Island Hospital Institutional Animal Care and Use Committee (AWC 5020-17).

## Author's Note

Systemic fungal infections such as those caused by Candida *sp*. are a frequent cause of nosocomial infections and are particularly problematic in patients maintained in the surgical ICU for extended periods of time. Neutrophils are the primary line of host defense against invasive Candida infections, and a better understanding of neutrophil-Candida interactions is therefore critical for improving patient outcomes with bloodstream disease. We show the release of the anti-candidal enzyme chitotriosidase (Chit1) can negatively affect the immune response against systemic candidiasis. Also, Chit1 enzymatic end-products can interfere with necessary leukocyte functions leading to an inefficient anti-fungal response. These findings implicate Chit1 inhibition as a target for pharmaceutical intervention against Candida infections.

## Author Contributions

NS and RT are co-first authors and contributed equally to experiments within this manuscript. C-SC provided histological analysis. C-ML, JE, and CL provided knockout mice and critical review of manuscript. BL authored the manuscript. All authors contributed to the article and approved the submitted version.

## Conflict of Interest

The authors declare that the research was conducted in the absence of any commercial or financial relationships that could be construed as a potential conflict of interest.

## Supplementary Material

Supplementary Figure 1 Control for Figure 1. No inherent fluorescence detectable in all treatment groups used in Figure 1 in the absence of (4-MU-DAC) substrate. Graph show representative example of experiment reproduced using three independent donors (*n* = 3).

Supplementary Figure 2 Chitotriosidase activity can be detected from plasma obtained from experimental animals. Chit1 activity of plasma was measured by the hydrolysis of 4-Methylumbelliferyl β-D-N,N′-diacetylchitobioside (4-MU-DAC) releasing fluorescent 4-MU. Serum from Wild type, lactose-treated and chitobiose treated mice showed significant activity at 1 h compared to substrate alone, while plasma obtained from Chit1 knockout mice showed no significant activity (^*^*P* < 0.05 experimental plasma vs. substrate alone, *n* = 7 mice/experimental group).

Supplementary Figure 3 Dose curve of chitobiose and soluble β-glucan on neutrophil clustering response on fibronectin and immobilized β-glucan. **(A)** Neutrophils plated on fn+β-glucan with increasing amounts of chitobiose show a dose-responsive reduction in cluster size corresponding with concentration of chitobiose. **(B)** Chitobiose induced reduction of cluster size was reversed in a dose-responsive manner by the addition of soluble β-glucan. Fibronectin with either chitobiose or soluble β-glucan alone did not induce neutrophil clustering. We chose 5 mM chitobiose and 960 μg/ml β-glucan for experiments.

Supplementary Figure 4 General location of image acquisition for quantitative analysis. Red outline indicates general area of quantitative (20X) image placement to include areas of active infection, while black outline shows relative location of included representative 4X images to show Ly6G staining distribution.
