# Supplementary figures and images for: Chitotriosidase Activity Is Counterproductive in a Mouse Model of Systemic Candidiasis
Source: Front Immunol. 2021 Mar 16;12:626798. doi: 10.3389/fimmu.2021.626798 (PMC8007879; doi:10.3389/fimmu.2021.626798)

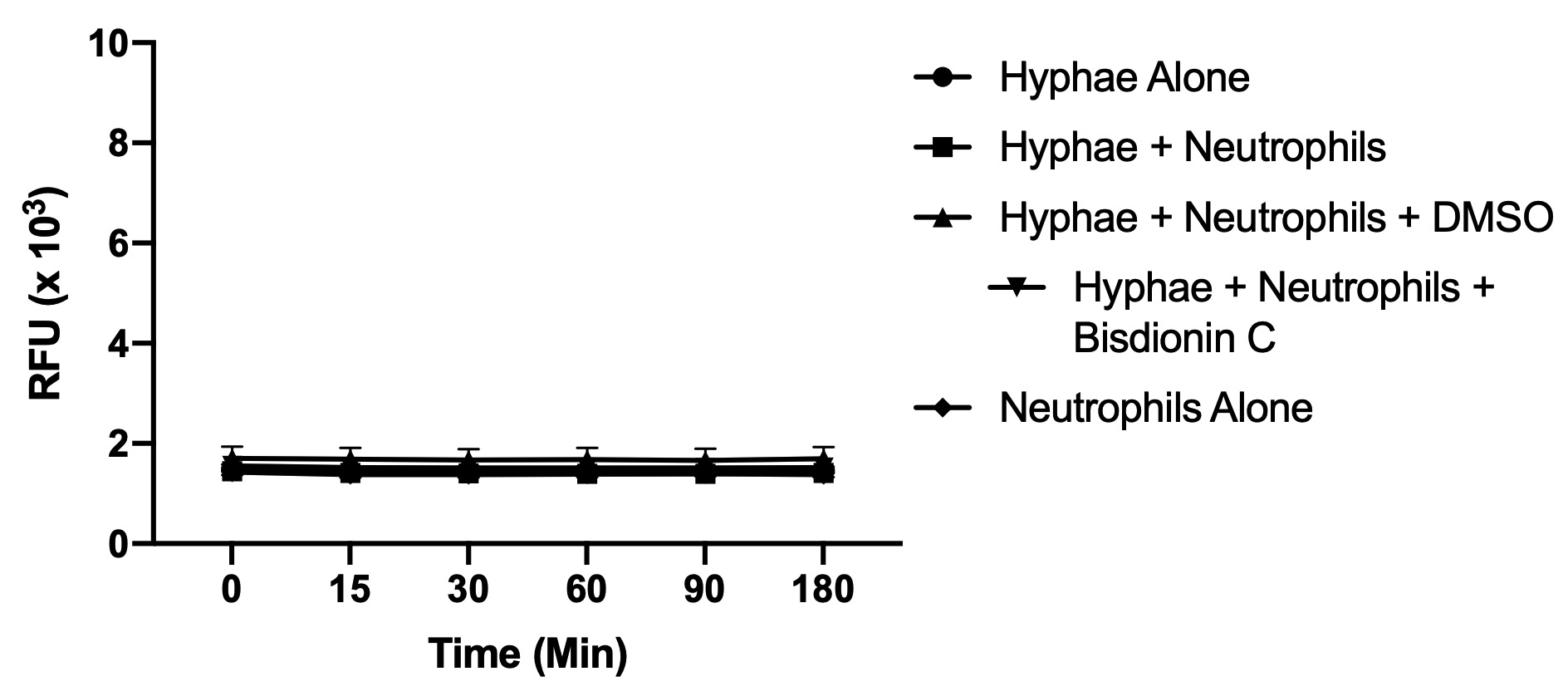

Supplement: Supplementary Figure 1 — Control for Figure 1. No inherent fluorescence detectable in all treatment groups used in Figure 1 in the absence of (4-MU-DAC) substrate. Graph show representative example of experiment reproduced using three independent donors (n = 3). [file Image_1.jpg]

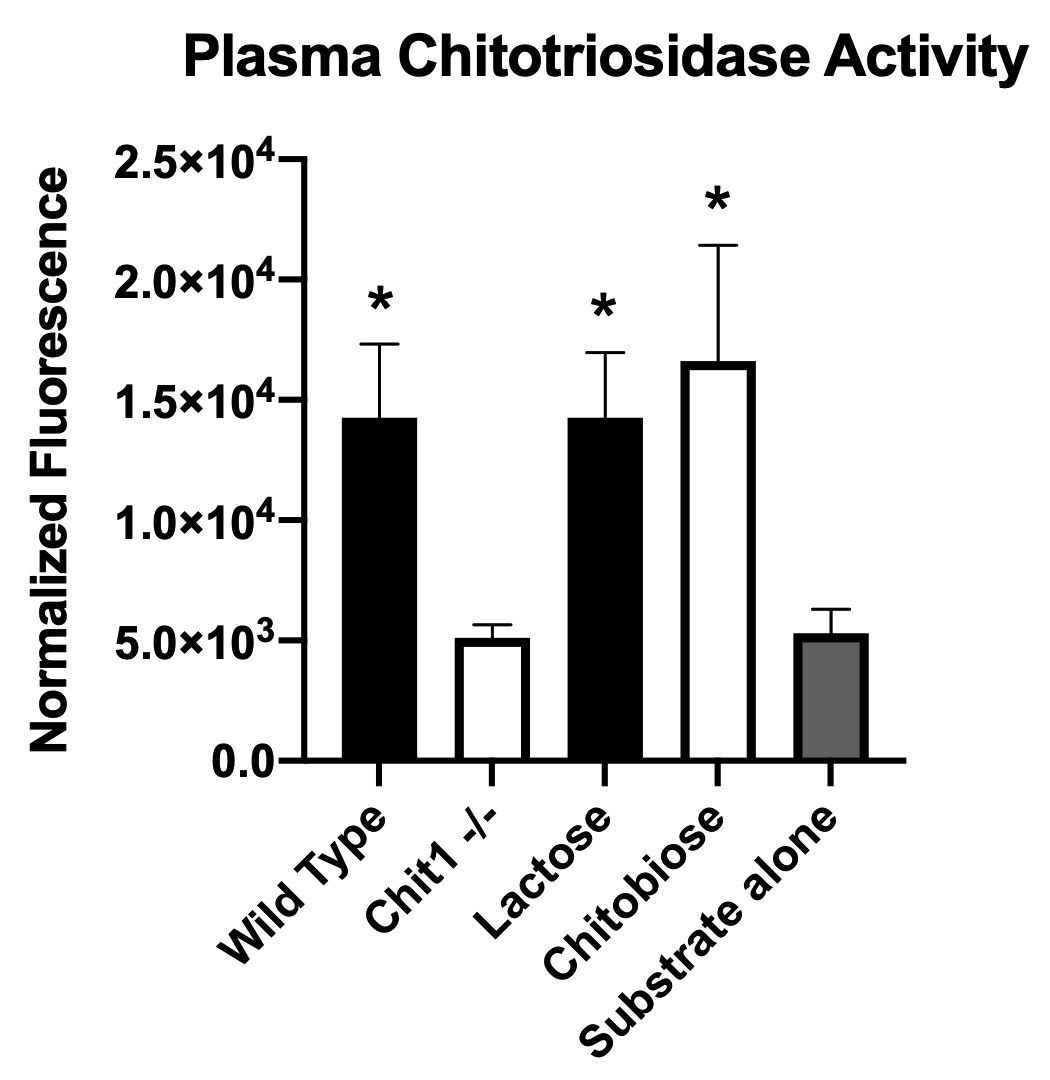

Supplement: Supplementary Figure 2 — Chitotriosidase activity can be detected from plasma obtained from experimental animals. Chit1 activity of plasma was measured by the hydrolysis of 4-Methylumbelliferyl β-D-N,N′-diacetylchitobioside (4-MU-DAC) releasing fluorescent 4-MU. Serum from Wild type, lactose-treated and chitobiose treated mice showed significant activity at 1 h compared to substrate alone, while plasma obtained from Chit1 knockout mice showed no significant activity (*P < 0.05 experimental plasma vs. substrate alone, n = 7 mice/experimental group). [file Image_2.jpg]

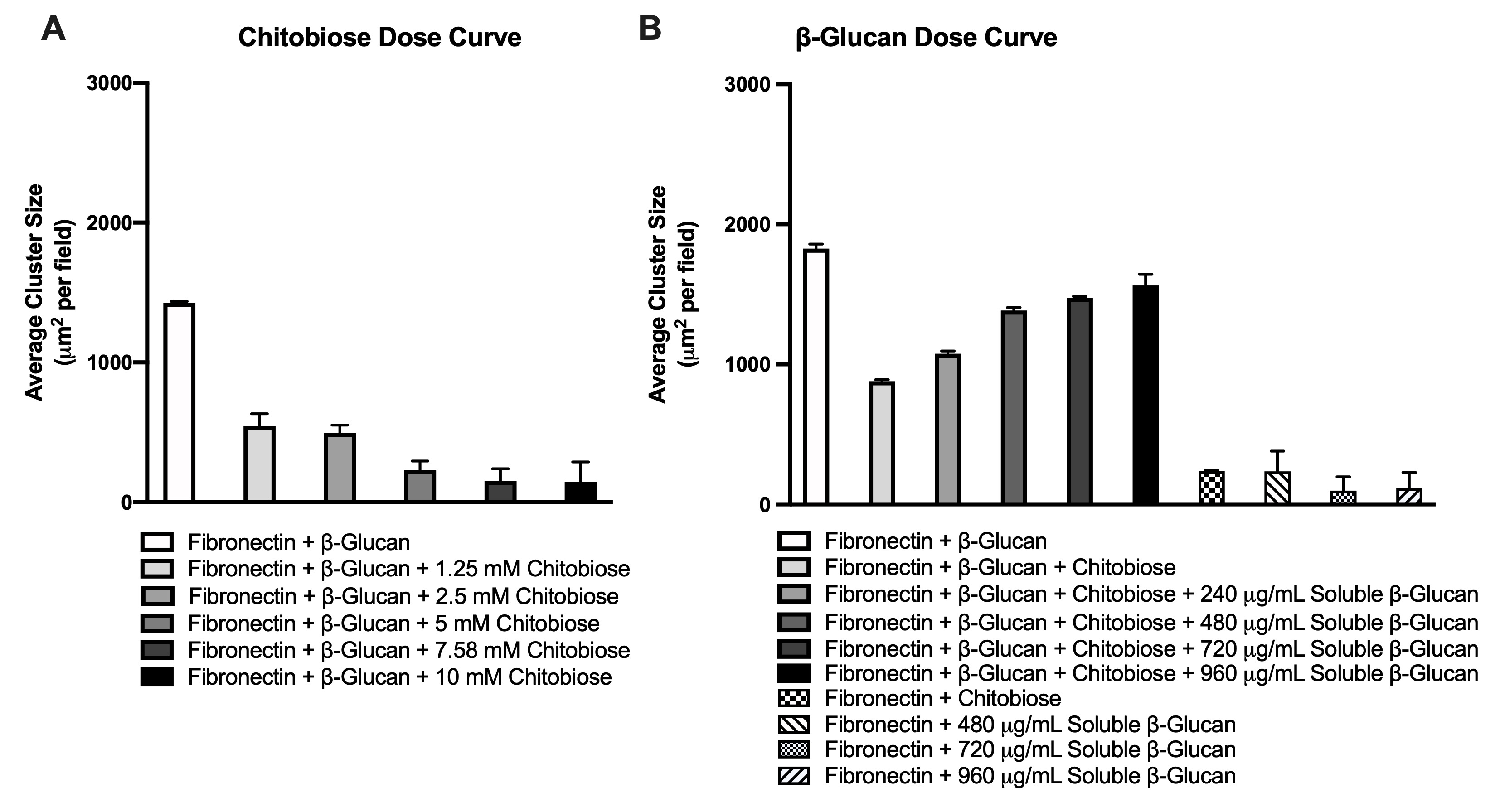

Supplement: Supplementary Figure 3 — Dose curve of chitobiose and soluble β-glucan on neutrophil clustering response on fibronectin and immobilized β-glucan. (A) Neutrophils plated on fn+β-glucan with increasing amounts of chitobiose show a dose-responsive reduction in cluster size corresponding with concentration of chitobiose. (B) Chitobiose induced reduction of cluster size was reversed in a dose-responsive manner by the addition of soluble β-glucan. Fibronectin with either chitobiose or soluble β-glucan alone did not induce neutrophil clustering. We chose 5 mM chitobiose and 960 μg/ml β-glucan for experiments. [file Image_3.jpg]

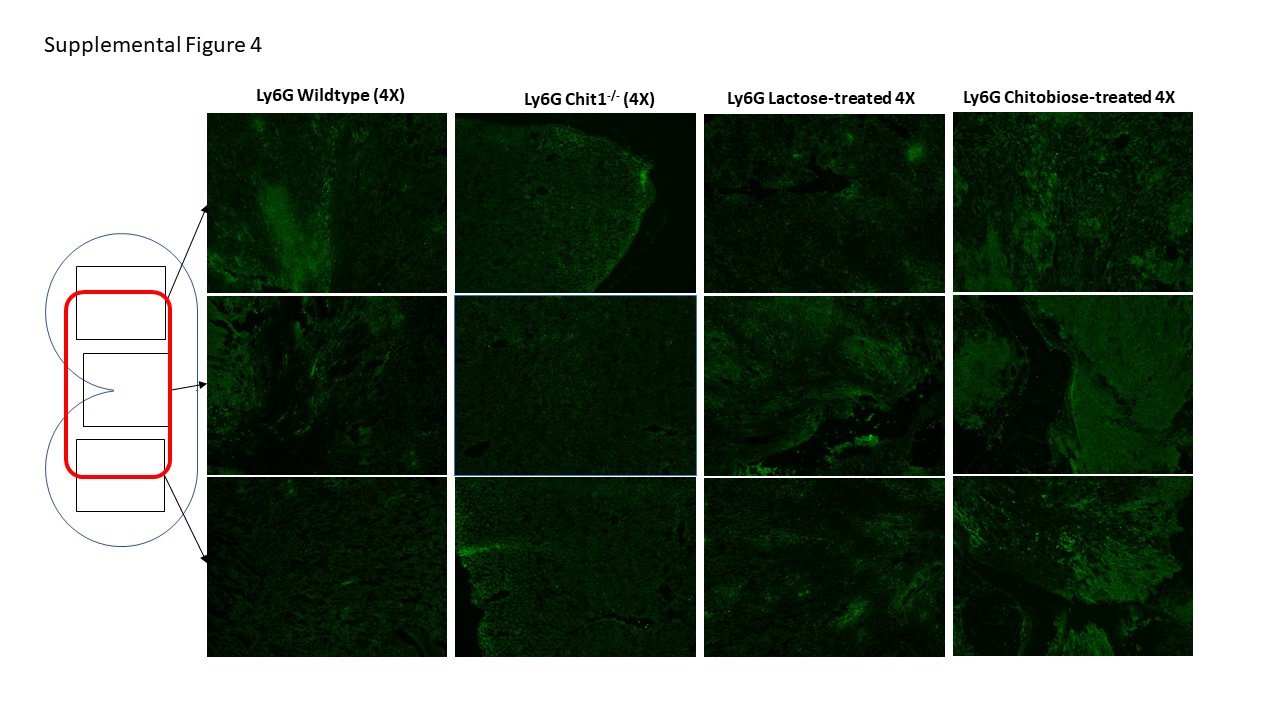

Supplement: Supplementary Figure 4 — General location of image acquisition for quantitative analysis. Red outline indicates general area of quantitative (20X) image placement to include areas of active infection, while black outline shows relative location of included representative 4X images to show Ly6G staining distribution. [file Image_4.jpg]
